# Changes in schizophrenia symptoms, tryptophan metabolism, neuroinflammation and the GABA-glutamate loop: A pilot study

**DOI:** 10.4102/sajpsychiatry.v31i0.2407

**Published:** 2025-04-14

**Authors:** Estmia Van der Walt, Christiaan B. Brink, Esmé Jansen van Vuren

**Affiliations:** 1 Centre of Excellence for Pharmaceutical Sciences, Faculty of Health Sciences, North-West University, Potchefstroom, South Africa; 2 Hypertension in Africa Research Team, Faculty of Health Sciences, North-West University, Potchefstroom, South Africa; 3 South African Medical Research Council: Unit for Hypertension and Cardiovascular Disease, North-West University, Potchefstroom, South Africa

## Introduction

Schizophrenia, a severe psychiatric disorder characterised by psychosis, imposes a significant disability burden, particularly with acute episodes.^1^ In sub-Saharan Africa, schizophrenia prevalence has risen by 126% in recent decades,^2^ yet limited data exists, as only 0.001% of global trial participants are recruited from Africa.^3^ Ethnic and cultural differences affect treatment responses,^4^ making biomarkers crucial for predicting outcomes.

Several models, as reviewed by Stahl,^5^ suggest that abnormalities in dopaminergic, serotonergic and glutamatergic signalling contribute to schizophrenia. Dysregulation of the gamma-aminobutyric acid (GABA)-glutamate loop is thought to contribute to both positive and negative symptoms.^6^ Glutamate neurons activate N-methyl-D-aspartate receptors (NMDARs) in the prefrontal cortex (PFC), which influence GABA interneurons and secondary glutamate neurons that regulate dopamine, noradrenaline and serotonin (5-HT) in the ventral tegmental area (VTA), limbic system and striatum. Failure to inhibit this loop can lead to excessive dopamine release in the VTA and mesolimbic system, causing positive symptoms, while hyperactivation of secondary glutamate neurons can increase GABA release in the PFC, inhibiting dopamine and 5-HT in the mesocortical system, resulting in negative symptoms.^7^ These theories are based on neuroimaging studies and animal models, but the exact causes of schizophrenia remain unclear.^8,9^

While traditional theories of schizophrenia focus on dopamine and glutamate signalling, newer research explores the redox-immune-inflammatory hypothesis, linking brain changes with immune and redox system dysfunctions.^10,11,12^ The presence of inflammatory markers in the periphery of patients suggests that chronic inflammation underlies schizophrenia.^13,14^ Pro-inflammatory signals activate microglia, triggering anti-inflammatory cytokines and brain-derived neurotrophic factor (BDNF) production to promote tissue repair.^15^ The immune system’s interaction with tryptophan metabolism is also implicated.^16^ In pro-inflammatory conditions, tryptophan – an essential amino acid and the primary precursor of 5-HT – is increasingly metabolised into kynurenine,^17,18^ generating neuroactive metabolites with opposing effects namely kynurenic acid (KYNA) and quinolinic acid (QUIN).^18^ Although these pathways are of significant interest in schizophrenia research, findings remain inconsistent across populations,^19,20^ leaving their roles in symptomatology unclear.

This pilot study therefore explored whether biomarkers related to tryptophan metabolism, neuroinflammation and the GABA-glutamate loop are associated with changes in schizophrenia and psychotic symptoms after 6 weeks of treatment in South African individuals with schizophrenia.

## Methods

### Study design and assessments

Twenty-eight black African patients with a diagnosis of schizophrenia (DSM-5 criteria) were recruited upon admission to a psychiatric hospital in the Dr Kenneth Kaunda District, North West province, South Africa, between January 2021 and June 2022. Patients were either newly diagnosed (first-episode psychosis) or had relapsed and had been antipsychotic free (i.e. did not receive any antipsychotics at dosages indicated for antipsychotic effects for at least 6 months prior to admission). Exclusion criteria included non-African descent, neurological disorders, head trauma, pregnancy, lactation, tympanum temperatures >37°C, substance abuse and tested positive for coronavirus disease 2019 (COVID-19). Patients exhibiting destructive, aggressive, or violent behaviour, as well as those who explicitly expressed dissent (indicating they did not wish to participate or refusal to have their blood drawn) were also excluded from the study. Fifteen patients met the inclusion criteria. Participation was voluntary, and independent psychiatrists assessed participants’ capacity to provide informed consent. Participants provided informed consent; those unable to do so provided assent. After the 6-week follow-up period, patients who initially provided assent were asked to provide delayed informed consent. Participants could withdraw at any time without explanation. Following baseline data collection, psychiatrists initiated individualised treatment based on hospital protocols. After 6 weeks, patients were followed up as inpatients or outpatients.

Psychiatrists assessed psychiatric symptom severity at baseline and follow-up using the Positive and Negative Syndrome Scale (PANSS). Fasting blood samples (10 mL) were collected at baseline and follow-up in serum-separating tubes via venepuncture before 09:00 to minimise diurnal variation. The samples were kept at room temperature for 30 min in a closed container during transport and upon arrival at the laboratory. Afterwards, they were centrifuged, aliquoted into cryovials and stored at −80°C in biofreezers following standardised procedures. Serum biomarkers, including tryptophan, kynurenine, KYNA, QUIN, 5-HT, 5-hydroxyindoleacetic acid (HIAA), dopamine, 3-methoxytyramine (MT), norepinephrine, 3,4-dihydroxyphenylacetic acid (DOPAC), homovanillic acid, glutamate and GABA, were measured using a validated liquid chromatography-mass spectrometry (LCMS) procedure.^21^ Briefly, serum was treated with equal volumes of a specified internal standard solution, after which protein precipitation was induced, and the samples were centrifuged according to specified standards. Thereafter, the supernatant was injected into the LCMS system. Tryptophan and metabolites thereof were detected through the Ultivo Triple Quadrupole LC-MS system, which is controlled by the MassHunter software. High-sensitivity enzyme-linked immunosorbent assay (ELISA) kits (catalogue numbers HS600C, HS100C and HSTA00E) were used to measure interleukin (IL)-6, IL-10 and tumour necrosis factor (TNF)-alpha (α), respectively. For BDNF, a sandwich ELISA (catalogue number DBNT00) was used. These kits are designed for the enzyme-linked detection and quantification of antibodies or antigens. All samples were analysed in duplicate following the manufacturer’s protocols (R&D Systems).

### Statistical analyses

Statistical analysis was conducted using IBM^®^ SPSS^®^ Statistics version 28 and GraphPad Prism version 8 for graphical illustrations. Descriptive statistics were calculated for clinical and demographic variables. Normality was assessed with QQ-plots, and non-Gaussian variables (KYNA, 5-HT, MT, glutamate, GABA, BDNF, IL-6, IL-10) were logarithmically transformed. Paired sample *t*-tests assessed changes over time and percentage changes over time (%Δ) were calculated as: ([follow-up−baseline]/baseline) × 100. Backward stepwise regression analyses determined associations between %Δ of PANSS scores (total, positive, negative, general) and baseline or %∆ biomarker levels. Significance was set at *p* ≤ 0.05, with age and socio-economic status as confounders. Sensitivity analyses were performed by substituting age and socio-economic status for body weight, respectively, to avoid model overfitting. However, the inclusion of body weight as confounder did not influence the outcome of the results and was therefore not included in the final models. To lower the false discovery rate of statistically significant relationships, we used the Benjamini-Hochberg procedure to calculate the corrected level of significance (reported as q-values). To address multicollinearity in our regression models, we ensured that all variance inflation factor (VIF) values were below 5.00.

### Ethical considerations

Ethical clearance to conduct this study was obtained from the North-West University Health Research Ethics Committee (No. NWU-00929-19-A1) and the North West Provincial Department of Health Research Committee.

## Results

This pilot study sample of 15 patients (12 men, three women), aged 22–35 years (mean 35 years), is shown in Table 1. Follow-up data were obtained for 53.5% (8/15) of the sample, all men. Socio-economic data were available for only nine patients, of these 53.3% classified as a low socio-economic status.

**TABLE 1:** Baseline demographic and clinical characteristics of the study sample (*N* = 15).

Variables	*n*	%	Mean	± 95% CI	Median	IQR
**Sociodemographic**						
Age	-	-	34.64	28.56; 40.71	33.00	12
Sex						
Male	12	80.0	-	-	-	-
Female	3	20.0	-	-	-	-
SES						
Low	8	53.3	-	-	-	-
Middle	1	6.7	-	-	-	-
Body weight	-	-	64.25	55.29; 73.20	65.00	17
**Psychiatric symptoms**						
PANSS total	-	-	82.00	65.17; 98.83	88.50	25
PANSS positive	-	-	25.00	20.05; 29.95	27.00	13
PANSS negative	-	-	20.85	14.09; 27.61	17.00	16
PANSS general	-	-	42.46	35.95; 48.98	43.00	13

CI, confidence interval; IQR, interquartile range; SES, socio-economic status; PANSS, Positive and Negative Syndrome Scale.

**Figure 1:**
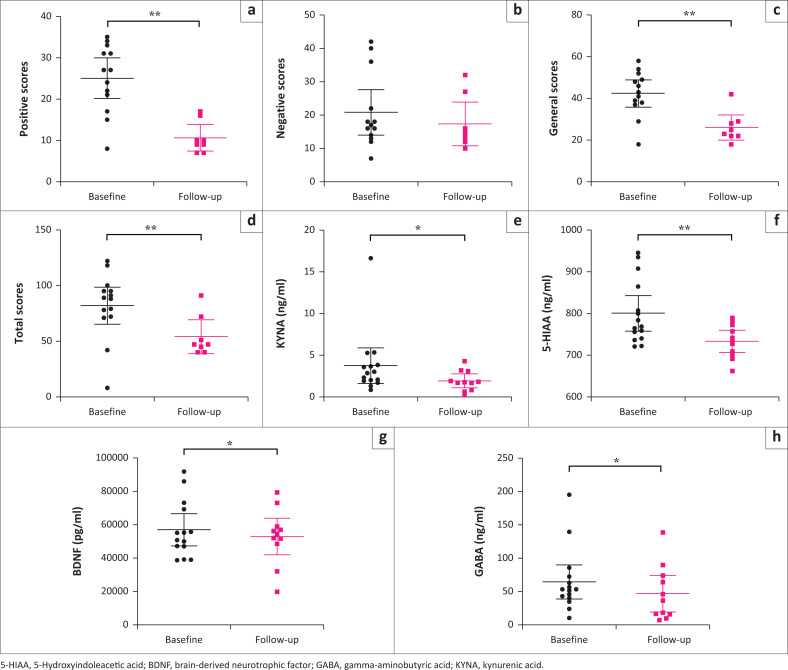
(a-d) Changes in psychiatric symptom severity; (e-f) biomarkers of tryptophan metabolism; (g) BDNF; (h) GABA over a six-week period. Statistical significance indicated by: *, *p* ≤ 0.05; **, *p* ≤ 0.001. Error bars represent 95% confidence interval.

Paired *t*-tests revealed significant decreases in PANSS positive, general and total scores over 6 weeks (*p* ≤ 0.001), but no changes in PANSS negative scores (Figure 1a–d). In Figure 1e–h, reductions are shown for KYNA (*p* = 0.04), 5-HIAA (*p* = 0.004), BDNF (*p* = 0.04) and GABA (*p* = 0.02). No changes were observed for tryptophan, kynurenine, QUIN or other biomarkers including 5-HT, dopamine, 3-MT, norepinephrine, DOPAC, homovanillic acid, glutamate, IL-6, IL-10 or TNF- α.

Associations between %∆ in PANSS scores with baseline and %∆ biomarkers are shown in Table 2. Initial significant associations shown between %∆PANSS total score with baseline KYNA (*p* = 0.015), %∆PANSS positive score with baseline KYNA (*p* = 0.046), %∆PANSS general score with baseline BDNF (*p* = 0.023) and %∆PANSS negative score with baseline glutamate (*p* = 0.027) and % ∆ kynurenine (*p* = 0.020) were lost after calculation of the corrected level of significance.

**TABLE 2:** Independent associations between Positive and Negative Syndrome Scale scores, brain-derived neurotrophic factor and biomarkers of tryptophan metabolism and the GABA-glutamate loop in different models.

Biomarkers	%Δ PANSS total score	%Δ PANSS positive score	%Δ PANSS negative score	%Δ PANSS general score
b_KYNA	Adj *R*^2^ = 0.59 0.81 (0.22; 1.40)^NS^	Adj *R*^2^ = 0.43 0.72 (0.02; 1.41)^NS^	-	-
b_glutamate	-	-	Adj *R*^2^ = 0.52 0.77 (0.13; 1.41)^NS^	-
b_BDNF	-	-	-	Adj *R*^2^ = 0.63 -0.90 (-1.61; -0.19)^NS^
b_5-HIAA	-	Adj *R*^2^ = 0.49 0.75 (0.09; 1.41)*	-	-
b_5-HT	-	-	-	Adj *R*^2^ = 0.87 0.94 (0.60; 1.28)**
% Δ kynurenine	-	-	Adj *R*^2^ = 0.61 0.90 (0.21; 1.59)^NS^	-
% Δ QUIN	-	-	Adj *R*^2^ = 0.71 -0.89 (-1.74; -0.03)*	-
% Δ 5-HIAA	-	Adj *R*^2^ = 0.46 -0.73 (-1.41; -0.05)*	-	-
% Δ GABA	-	-	Adj *R*^2^ = 0.54 1.11 (0.21; 2.00)*	-

%Δ, changes; 5-HIAA, 5-Hydroxyindoleacetic acid; 5-HT, serotonin; b, baseline; BDNF, brain-derived neurotrophic factor; GABA, gamma-aminobutyric acid; KYNA, kynurenic acid; NS, not significant; QUIN, quinolinic acid; PANSS, Positive and Negative Syndrome Scale.

Data is presented as standardised β (95% CI). Superscript symbol shows the corrected level of significance using the Benjamini-Hochberg procedure (reported as *q*-values):

* *q* ≤ 0.05,

** *q* ≤ 0.01. Models are adjusted for age and socio-economic status.

Significant associations remained between %∆PANSS positive score with baseline 5-HIAA (*p* = 0.032, *q* = 0.035) and inversely with %∆5-HIAA (*p* = 0.039, *q* = 0.040), %∆PANSS general score with baseline 5-HT (*p* < 0.001, *q* = 0.005), as well as %∆PANSS negative score with %∆GABA (*p* = 0.024, *q* = 0.025) and inversely with %∆QUIN (*p* = 0.046, *q* = 0.045). No associations were revealed for any of the other biomarkers including tryptophan, dopamine, 3-MT, norepinephrine, DOPAC, homovanillic acid, IL-6, IL-10 or TNF-α.

## Discussion

This study assessed peripheral biomarkers and psychiatric symptom changes after 6 weeks of antipsychotic treatment in a cohort of South Africans with schizophrenia. Significant improvements were observed in the positive, general and total PANSS scores, potentially linked to tryptophan metabolism. However, negative scores showed no improvement, which may be related to tryptophan metabolism and the GABA-glutamate loop.

### Psychiatric symptom changes

Distinct treatment response patterns have been observed in first-episode schizophrenia. Regarding negative symptoms, some of our patients may not have responded to treatment by the 6-week follow-up. Even in studies with longer follow-ups (8 weeks and 18 months), no significant improvement in negative symptom scores was found, despite improvement in other PANSS scores.^22,23^ This lack of improvement may be because of the limited efficacy of antipsychotics and antidepressants, prescribed in our study, for negative symptoms, which tend to improve more gradually than positive symptoms. Additionally, only two patients in our study sample were treated with selective serotonin reuptake inhibitors (data not shown), indicated for negative symptoms, and the varied treatments prescribed limit our ability to comprehensively explain the role of treatment in this regard.

### Tryptophan metabolism: The kynurenine pathway

The kynurenine pathway produces neuroactive substances like QUIN and KYNA, which have neuroprotective and neurodegenerative effects. Kynurenic acid, an NMDAR antagonist, can be neuroprotective but may disrupt neurotransmission at high levels by inducing NMDAR hypo-functioning. Our study does not support KYNA’s neuroprotective role, as no significant associations were found between KYNA changes and any of the PANSS scores, despite the significant reductions observed for KYNA. This contrasts with another study, which reported a correlation between KYNA levels at admission with changes in PANSS positive scores.^24^ We found an association between the PANSS negative score with changes in QUIN. At excitotoxic concentrations, QUIN is not only known to activate various pro-inflammatory cytokines but also serves as an NMDAR agonist causing excitotoxic neurodegeneration.^18^ Therefore, the lack of significant changes in QUIN over 6 weeks may explain the unimproved negative symptoms.

### Tryptophan metabolism: The serotonin pathway

Decreases in the PANSS general score associated with baseline 5-HT levels. Somewhat consistent with our findings, 5-HIAA, the primary metabolite of 5-HT, measured post-mortem correlated with general schizophrenia symptoms assessed perimortem.^25^ However, as there were no significant changes in 5-HT levels or associations between the PANSS general score and 5-HIAA changes, we cannot conclude that alterations in 5-HT metabolism influenced the improvement in general symptoms.

Increases in 5-HT transmission, particularly in the striatum, have been suggested to contribute to positive symptoms in animal models of schizophrenia,^9^ which is supported by the findings in our study where decreases in the PANSS positive score associated with baseline 5-HIAA levels and inversely with decreases in 5-HIAA levels.

### Gamma-aminobutyric acid-glutamate loop

Changes in the PANSS negative score associated with decreases in GABA. Similarly, individuals with schizophrenia that did not respond to antipsychotic treatment had lower GABA levels in the anterior cingulate cortex compared to healthy controls.^26^ Negative symptoms in schizophrenia may arise when primary glutamate neurons activate GABA interneurons, leading to hyperactivation of secondary glutamate neurons and inhibiting mesocortical dopamine and 5-HT activity,^7^ as described previously. As GABA decreased over 6 weeks, this may explain the persistence of negative symptoms in our study.

### Limitations

The primary limitation of this study is the small sample size, which may increase the risk of Type I errors and reduce the reliability of the results. As this was a pilot exploratory study, the findings should be interpreted with caution, recognising that they serve as a preliminary step for generating hypotheses rather than drawing definitive conclusions. Additionally, as participants were recruited from a single psychiatric hospital and ethnic group, this limits the generalisability to the broader South African population. It has been shown that an increase in body weight is related to the efficacy of antipsychotics and might predict general schizophrenia symptomatology improvement.^27^ Unfortunately, data on body weight following the treatment period was not available for this study, preventing us from assessing whether changes in body weight may have influenced our findings. While all procedures followed the hospital’s standardised protocols to minimise patient stress, we did not assess biomarkers or stress directly. Therefore, we cannot determine whether symptom improvement was linked to stress reduction as symptoms eased with treatment. While blood-based biomarkers may not directly reflect central nervous system (CNS) levels, they are often correlated with cerebrospinal fluid and brain tissue biomarkers in schizophrenia patients. The study offers valuable insights and hypotheses for future research in this under-investigated population.

### Conclusion

After 6 weeks of antipsychotic treatment, improvements were seen in PANSS total, positive and general sub-scores, along with decreases in KYNA, 5-HIAA, BDNF and GABA levels. It remains unclear if symptom relief was because of antipsychotics’ effects on tryptophan metabolism. The lack of significant reduction in negative symptoms may be linked to unchanged QUIN levels, as well as decreases in GABA. Further research is needed to clarify the role of these biomarkers in antipsychotic treatment response, which could guide treatment strategies, particularly in African populations.
